# The personalized cancer network explorer (PeCaX) as a visual analytics tool to support molecular tumor boards

**DOI:** 10.1186/s12859-023-05194-3

**Published:** 2023-03-08

**Authors:** Mirjam Figaschewski, Bilge Sürün, Thorsten Tiede, Oliver Kohlbacher

**Affiliations:** 1 Department of Computer Science, University of Tübingen, Tübingen, Germany; 2 Institute for Bioinformatics and Medical Informatics, University of Tübingen, Tübingen, Germany; 3 Institute for Translational Bioinformatics, University Hospital Tübingen, Tübingen, Germany

**Keywords:** Precision medicine, Personalized oncology, Clinical decision support, Gene drug interaction networks

## Abstract

**Background:**

Personalized oncology represents a shift in cancer treatment from conventional methods to target specific therapies where the decisions are made based on the patient specific tumor profile. Selection of the optimal therapy relies on a complex interdisciplinary analysis and interpretation of these variants by experts in molecular tumor boards. With up to hundreds of somatic variants identified in a tumor, this process requires visual analytics tools to guide and accelerate the annotation process.

**Results:**

The Personal Cancer Network Explorer (PeCaX) is a visual analytics tool supporting the efficient annotation, navigation, and interpretation of somatic genomic variants through functional annotation, drug target annotation, and visual interpretation within the context of biological networks. Starting with somatic variants in a VCF file, PeCaX enables users to explore these variants through a web-based graphical user interface. The most protruding feature of PeCaX is the combination of clinical variant annotation and gene-drug networks with an interactive visualization. This reduces the time and effort the user needs to invest to get to a treatment suggestion and helps to generate new hypotheses. PeCaX is being provided as a platform-independent containerized software package for local or institution-wide deployment. PeCaX is available for download at https://github.com/KohlbacherLab/PeCaX-docker.

**Supplementary Information:**

The online version contains supplementary material available at 10.1186/s12859-023-05194-3.

## Background

Cancer is typically caused by genomic alterations inducing unchecked cellular proliferation. In personalized oncology [1], molecular data (e.g., genomics) is used jointly with clinical data to stratify therapies and choose the therapy best-suited for a specific patient. Next Generation Sequencing (NGS) is widely used to find those genomic alterations, such as single-nucleotide variants (SNVs), copy number variations (CNVs), or gene fusions.

Based on this data, the typical analysis workflow is usually as follows:

1. The (cancer) genome of a patient is sequenced and the SNVs and CNVs are stored in a Variant Call Format (VCF) file.
2. Variants are annotated with their effect.
3. Usually only variants with a strong effect are considered.
4. The remaining variants are looked up in databases to identify driver genes and to find drugs associated with these potential targets.
5. If no drug can be found for this specific variant or it is not applicable for the patient, pathways containing the related gene are considered to find druggable targets up- or downstream of the actionable variant.

This process of revealing drug-gene information based on the variant annotations and incorporating pharmacogenomics information which is an indicator of the effect of the genes on a patient’s drug response is called clinical annotation.

Numerous tools exist for displaying and storing the information of a VCF file in the common tab separated values format (step 1) and to filter the variants for given annotations (step 3), e.g., VCF-Miner [2], BrowseVCF [3], VCF-Explorer [4]. But only few applications include the analysis of the SNVs and CNVs and the annotation of the variant effect (step 2), e.g., VCF-Server [5]. Perera-Bel et al. offer the additional option to find drugs targeting the variants (step 4) but their method does not perform variant effect prediction (step 2) and is limited to a specific data structure [6]. It also lacks a graphical user interface (GUI). OncoPDSS performs steps 1 to 4 but it is a web-server which can be a data security issue. Therefore, OncoPDSS does not store the input or results of the analysis [7]. These are displayed in unsearchable tables focusing on information about available pharmacotherapies which can be downloaded as TSV files. But it does not give information on the pathway context of a gene. So far, this information has to be collected manually.

We present PeCaX (Personalized Cancer Network Explorer), an integrated application for personalized oncology workflows. PeCaX performs clinical variant annotation by processing SNVs and CNVs and identifying clinically relevant variants and their targeting therapeutics using ClinVAP [8]. Networks containing the connections between the driver genes and the genes in their neighborhood as well as drugs targeting genes in this network are created through the novel SBML4j [9] and they are visualized with the use of BioGraphVisart [10], developed specifically for PeCaX. Our user-friendly, web-based graphical user interface does not only interactively display the report generated by ClinVAP and the networks with a few clicks, but adds web links to external gene and drug databases and gives the option to take notes which are stored in PeCaX along with the information presented in the tables and networks. This allows the user to interactively work on and present the results, e.g. in a Molecular Tumor Board (MTB) where clinicians of different areas meet, especially researchers in personalized oncology. The report can be downloaded as PDFs. In addition, the networks are available for download in publication-ready file formats (PNG, SVG and GraphML).

In contrast to many VCF-analysis and clinical decision support tools, PeCaX is a local application with a graphical user interface working on the user’s local machine avoiding data privacy issues arising from the use of cloud-based services.

## Implementation

### Technical overview

**Fig. 1:**
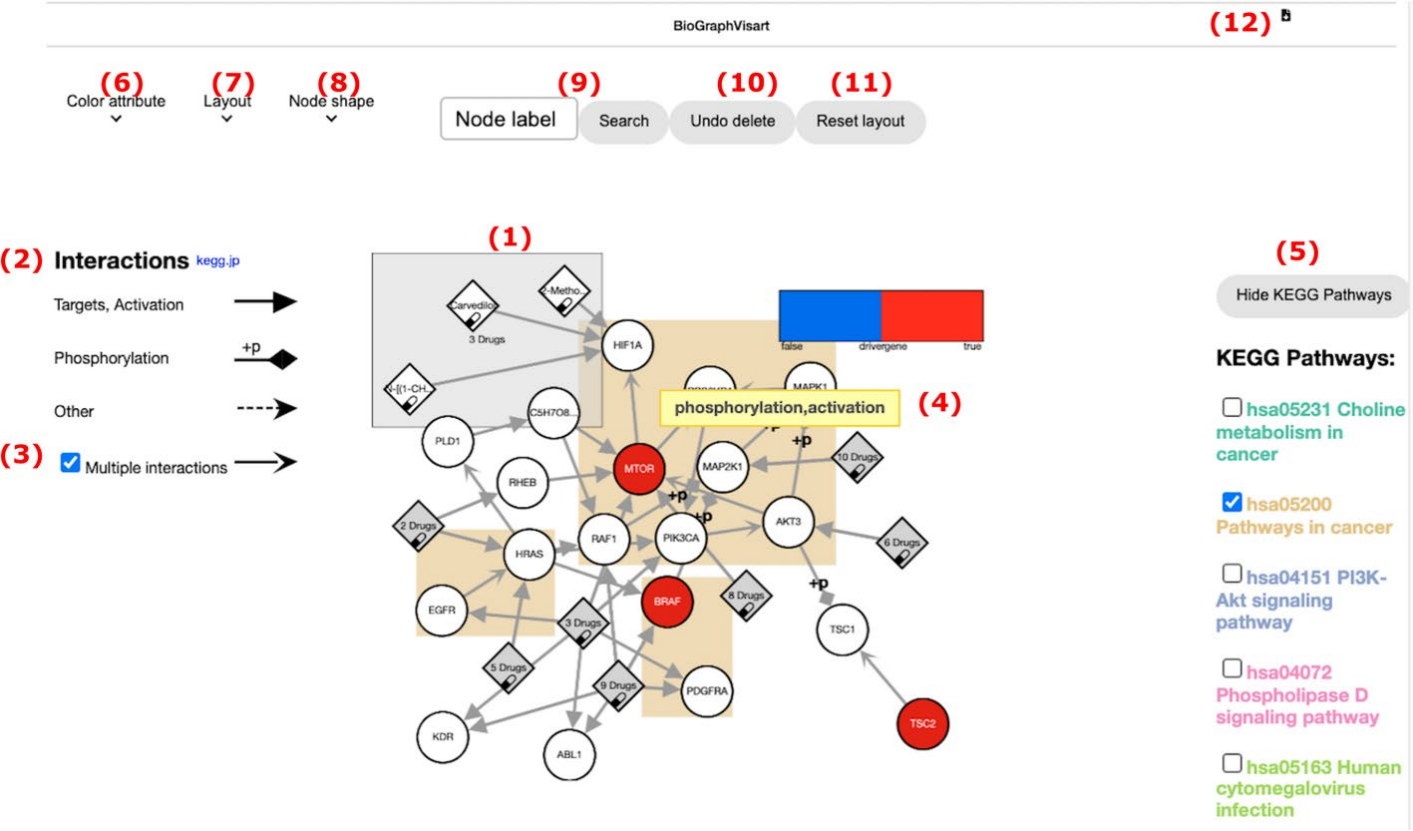
Architecture of PeCaX. Databases are indicated by the database icon

PeCaX is a service-oriented local application and has been built using the NuxtJS framework for its web-based front end and integrates several other local services developed by us via REST APIs (see Fig. 1). It was developed in close interaction with persons responsible for MTB case management, scientific analysis, case preparation and presentation at the University Hospital Tübingen to ensure a user-friendly user interface. It supports concurrent use and works on any modern web browser independent of the operating system. Its design as a web-service allows access from browsers not running on the same machine as the service. Sensitive data is only processed on the machine PeCaX is installed on, not the machines that access it with the GUI. It is easy to deploy via pre-built docker containers and easily integrated using docker compose. The individual docker containers and the communication via REST APIs allow to update the services individually without the need to setup everything from the start.

### Clinical variant annotation

PeCaX relies on VCF files for information on SNVs and TSV files for optional CNV information. Examples are available in the Additional file 1: Sect. 2.1.1. The validity of files to be uploaded is checked by the filename extension (.vcf,.tsv). If a uploaded file contains semantic or syntactic errors, the analysis process is aborted and the user is notified that the input file is corrupt.

PeCaX integrates ClinVAP [8] to create a case report by processing variants using functional and clinical annotations of the genomic aberrations observed in a patient. ClinVAP employs Ensemble Variant Effect Predictor (VEP) [11] to obtain functional effects of the observed variants and filters them based on the severity of the predictions.

It also performs clinical annotation which reveals the driver genes, actionable targets and enriches them with their known therapeutic associations using an integrated knowledge-base from publicly available databases (e.g., COSMIC [12], CGI [13]) [8]. Moreover, it provides an option to filter the results based on the diagnosis type given as ICD10 code which was achieved by obtaining the gene-disease links from the background databases and mapping those diseases to their corresponding ICD10 codes. The mapping between the disease names from the databases and from ICD10 is done by matching their disease related features such as system, organ, histology type. As soon as the annotation is finished the variant files are deleted and PeCaX receives the resulting report as JSON file with information structured into five categories: known driver genes, drugs targeting the variants, therapeutics targeting the affected genes, cancer drugs targeting the mutated genes, and drugs with known adverse effects (Additional file 1: Sect. 2.3.1).

### Network generation

Cancer is a complex and heterogeneous disease typically caused by genomic alterations. Even a single mutation can modulate the complex interaction network of genes to cause cancer phenotypes. Since these mutations can occur in arbitrary genes, it is useful to understand the role of the altered genes in their physiological context, i.e., within the context of their regulatory networks. By examining the network neighborhood of an altered gene, potential new treatment approaches can be identified for patients without other treatment options (e.g., through targeted therapies). Examining the interplay of gene-drug interactions using networks give insights into the effect of an intervention, for example, for a patient resistant to a drug. If the altered gene is not a drug target or cannot be targeted because of drug resistance or intolerance of the patient, the genes up- or downstream of it might be suitable drug targets.

Thus, PeCaX sends the list of genes of each category to SBML4j [9] which is a service for persisting biological models and pathways in SBML format in a graph database (Additional file 1: Sect. 2.2). Its graph database is used to extract information on the local network context of each candidate gene. Based on cancer-related pathways from KEGG [14], PeCaX can thus infer which related genes are up- and downstream of a candidate gene with respect to gene regulation and signalling. Additionally, SBML4j gives information about drugs associated with any of the candidate as well as up-/downstream genes.

### Interactive graphical user interface

PeCaX provides a simple graphical user interface to upload variants and display the results. The report generated by ClinVAP is displayed in an interactive tabular form next to the networks generated by SBML4j.

For an easier analysis of the networks, they are visualized as network graphs using BioGraphVisart [10]. The goal of the network analysis is to not only see the individual component but also the local neighborhood crosstalk with known pathways, and nearby options for therapeutic intervention (druggable genes). BioGraphVisart is a web-based tool written in Javascript. It automates the layout of the network graph, the labeling of nodes (genes, drugs) and edges (interactions), the edge style for different interaction types, the node coloring according to easily modifiable node attributes, and the generation of legends. In addition, human genes and proteins can be grouped with respect to predefined pathways from KEGG.

### Data management

The user has to give a project name before starting analysis. This name is used to create a collection in the local database (ArangoDB) used for data management. By that, the user can perform multiple analyses gathered in one collection, e.g., different patients presented in one MTB session. Starting an analysis the uploaded data and set parameters are send via REST API to the ClinVAP container and a unique job id is generated to store the parameters. The uploaded data is only stored during the clinical annotation and afterwards removed. The results of the analysis as well as the ids of the networks generated with SBML4j are stored related to the job ID. The networks themselves are stored in the network database.

## Deployment

PeCaX can be installed locally on a personal computer or for groups of users in an access controlled intranet. Containerization enable convenient deployment without complex software installation and configuration.

## Results

### Overview of PeCaX

PeCaX is a comprehensive GUI-based clinical decision support tool that requires no programming knowledge. Users can perform clinical annotation and gene drug interaction network analysis via the interactive graphical interface. PeCaX provides data security as it comes in Docker containers and all analysis are performed on local infrastructure. It is supported by all modern web browsers across platforms. Hence, it is easily integrated into diagnostic and MTB workflows to investigate the relevance of single variants, complete cases or cohorts, e.g. from GWAS.

We provide a web page with example data for demonstration purpose only at https://pecax.informatik.uni-tuebingen.de.

### Data upload

To annotate and analyze a data set, PeCaX requires (local) submission of the data. All data is assigned to a specific *project*, a generic way of grouping data sets and results (e.g., one project per tumor board meeting or for one tumor entity). PeCaX requires one VCF file as input containing information on SNVs and (optionally) a TSV file containing information on CNVs. Details on format requirements and conventions are found in the Additional file 1: Sect. 2.1.1. In addition the assembly of the human reference genome used in the mapping of the sequencing data is required (both GRCh37 and GRCh38 are supported). The clinical variant annotation can be filtered by a pre-selected cancer diagnosis given as ICD10 code. After uploading, the data is automatically submitted to a local instance of ClinVAP. In order to ensure data privacy, the VCF files are removed from the containers after processing by ClinVAP. The results of the variant annotation are stored in the project database in JSON format together with associated metadata (e.g., project name and the job ID). The user can also upload a previously downloaded JSON file which will skip ClinVAP or can enter the job ID of an analysis executed previously to directly get to the analysis part of the UI (Additional file 1: Sect. 2.4). All job IDs of a given project are listed on a subpage where the user can select and delete them individually. Deletion of a job ID removes all information stored for this ID in the project database as well as the generated networks from the network database to ensure data privacy.

### Interactive visualizations

The results of the clinical variant annotation performed is structured into several sections, which are all rendered as interactive, responsive tables. The first section contains the list of known cancer driver genes along with the somatic mutations observed in the patient. The list of drugs with the evidence of targeting a specific variant of the mutated gene and the documented drug response for the given mutational profile are displayed in the second section. The third section contains information on somatic mutations in pharmaceutical target affected genes and consists of two tables: therapies that have evidence of targeting the affected gene and the list of cancer drugs targeting the mutated gene. The fifth section contains the list of drugs with known adverse effects. References supporting the results found are displayed in a sixth section and all the somatic variants of the patient with their dbSNP and COSMIC IDs are listed in the last section.

**Fig. 2:**
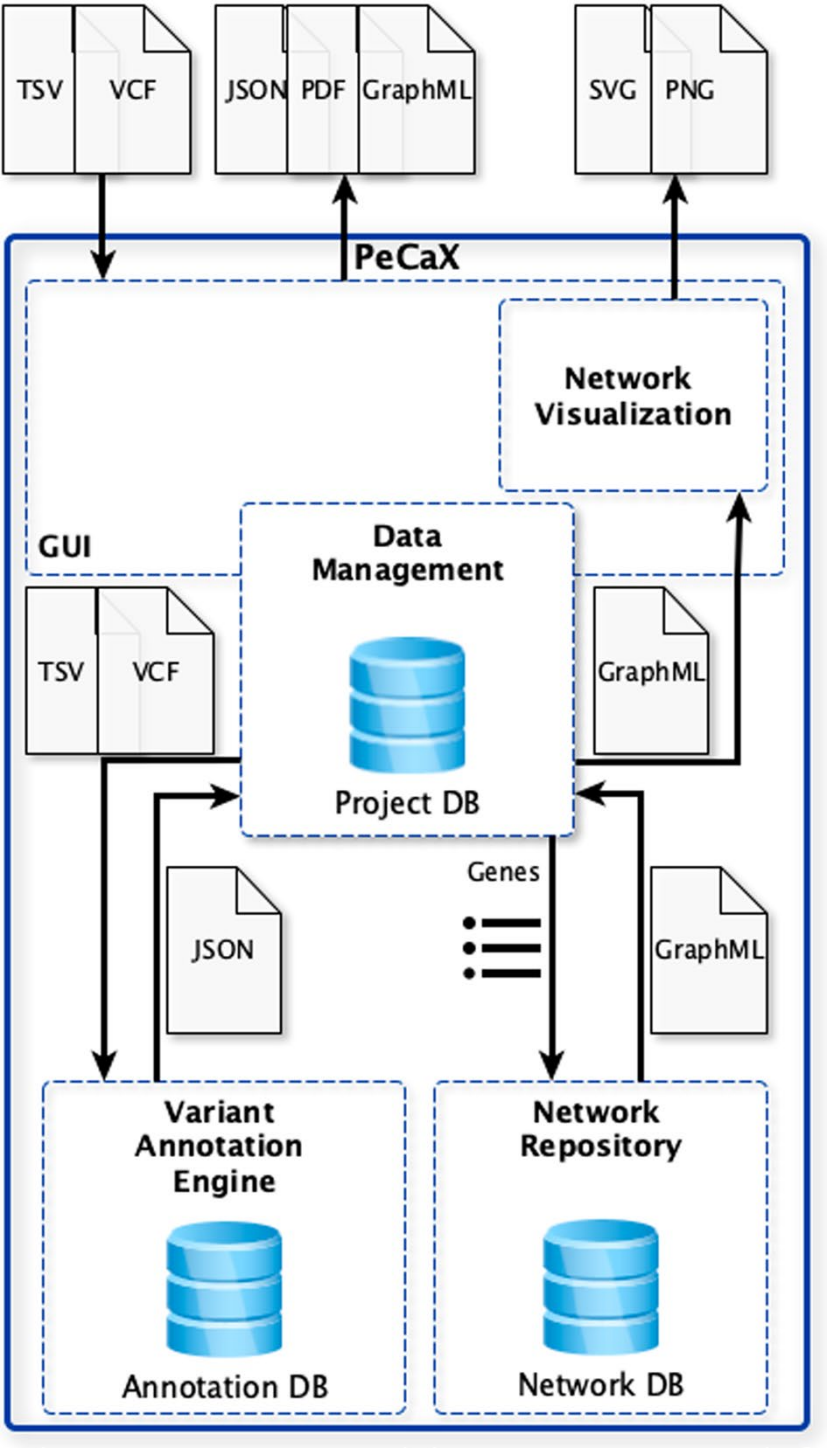
Interface for the interactive visualization of a table. **1** Filter table. **2** Sort table or hide/show column. **3** Highlight row across all tables by gene. **4** Genes and mutations are linked to external databases. **5** User can take notes which are saved. **6** Download table as PDF. **7** References directly link to their PubMed or clinicaltrials.gov entry

Figure 2 shows an example table of the results visualization. Each column of each table can be queried, filtered and sorted individually (Fig. 2(1)). The interactive table view supports a wide range of table operations in order to simplify navigation of the data, for example, hiding/showing columns (Fig. 2(2)), highlighting of rows across sections (Fig. 2(3)). For each gene listed, the tables contain links to various external data sources such as Uniprot [15], KEGG [14] or Ensembl [16]. The links can be accessed via the drop-down menu next to the gene symbol (Fig. 2(4)) and open in a separate browser tab or window. Likewise, the references given in the tables are directly linked to the web page of the related publication on PubMed or clinicaltrials.gov (Fig. 2(7)). At the end of each section, users can add notes to be stored along with the annotated data in the internal database (Fig. 2(5)). These notes can be used to record conclusions from the analysis of the data and can be downloaded together with the table as PDF (Fig. 2(6)).

**Fig. 3:**
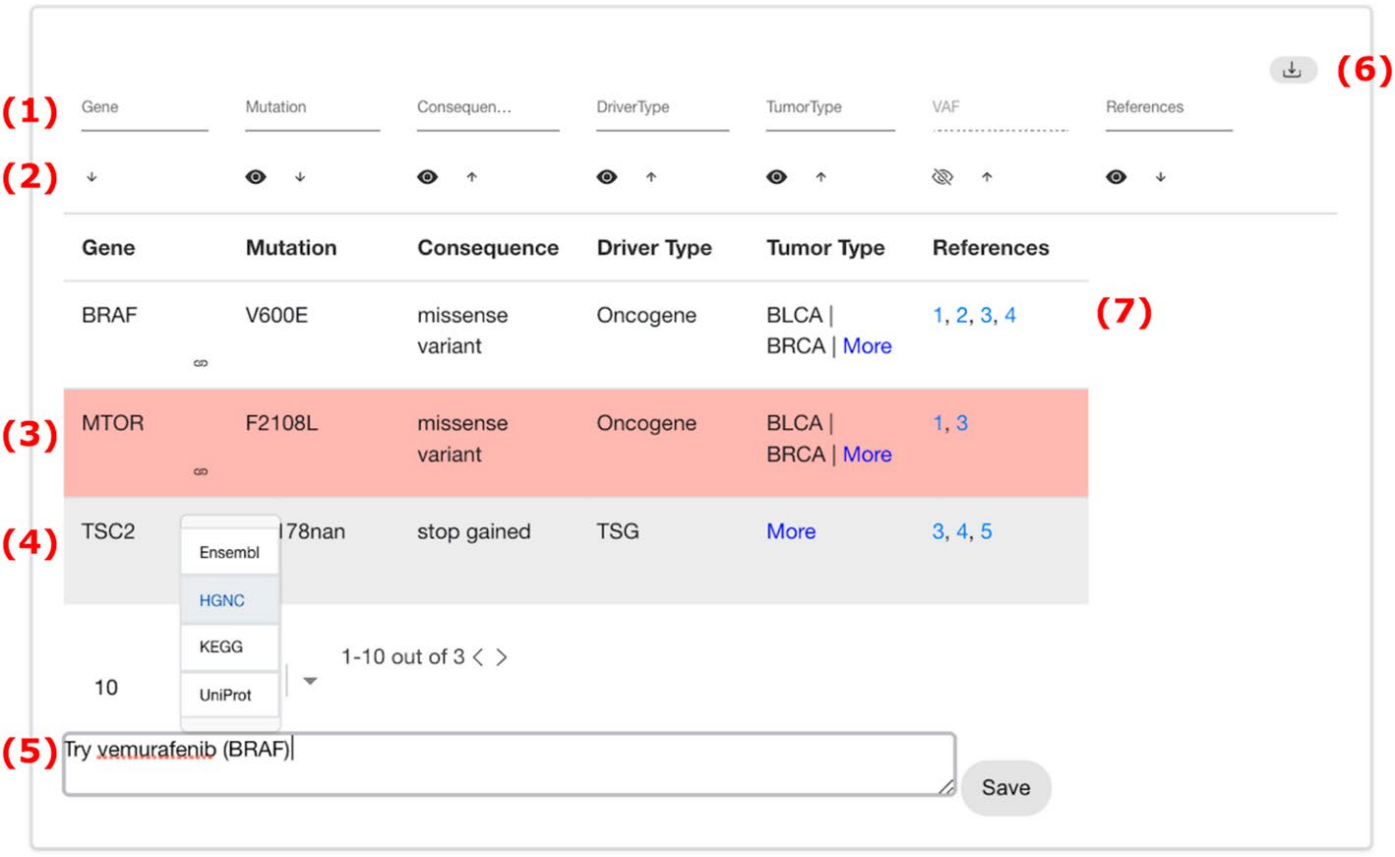
Interface for the visualization of a network. **1** Gene nodes have a circle shape, drug nodes a diamond shape. Multiple drugs targeting the same gene can be collapsed into one node. **2** Edge style legend. **3** Collapse/expand multiple edges between two nodes. **4** Moving the mouse over an edge displays the interactions. Moving it over a node displays the node label and secondary names. **5** Group nodes by KEGG pathways. **6** Selection of node color attribute. **7** Layout selection. **8** Highlight Boolean node attribute by node shape **9** Search a node by its label and highlight it. **10** Withdrawal of node deletion achieved by right click on a node. **11** The network itself and each individual node can be dragged. The layout can be reset. **12** Download of the visualization in the formats PNG, SVG

When at least one gene symbol of a table can be associated with an entry in the SBML4j database, a network for this table will be generated and is displayed next to it (see Fig. 3). The networks consist of nodes (genes, drugs) and edges (interactions). Genes found in the table are colored red and labelled with the gene symbol. Drugs associated with any of the genes in the network are represented as diamonds and are labelled with the drug name (Fig. 3(1)). If multiple drugs have the same gene target, they are merged into one node which is expandable in order to make the network representation visually more concise. Different interaction types (e.g., signaling, regulation) are depicted by different edge styles (Fig. 3(2)). If two nodes have multiple interactions, their edges are merged into one, which can be deactivated by the user (Fig. 3(3)). Since drug and gene names may become very long, they are shortened and moving the mouse over a node reveals the full node name. Edge types are treated in the same manner (Fig. 3(4)). The layout can be changed by the user based on five different layout types or the user can drag the nodes or the whole network manually to arrange them in the most informative layout (Fig. 3(7)). The nodes are searchable by the node label (Fig. 3(9)) and deletable including connected edges. Gene nodes can be grouped and highlighted by associated KEGG pathways (Fig. 3(5)). A mouse click on a drug node links to an overview page with links to external databases containing information on this drug such as Drugbank [17], HGNC [18], and PDB [19].

### Exporting tables and networks

The clinical variant annotation report including the stored user-created notes and manual annotations can be downloaded as a whole in PDF and JSON format or every table individually in PDF format (Fig. 2(6)). The gene drug interaction networks are available for download individually in the formats PNG, SVG (Fig. 3(12)) and GraphML.

### Performance

The processing time was evaluated using the example data provided in Additional file 1: Sect. 2.1.1. From submitting a VCF-file until the full report is displayed, PeCaX needs about 92*s* on average, see Table 1. The time needed for clinical annotation (45.48*s*) is less than for network generation (58.19*s*). For a VCF-file in combination with a related TSV-file, it takes on average about 352*s*. Here, too, network generation takes longer than clinical annotation. Overall, PeCaX needs 205*s* on average for the analysis of the data until the results are displayed. A detailed performance evaluation of the processing time for the example data is recorded in Additional file 1: Sect. 6. A detailed performance evaluation of ClinVAP and the results of a stress-test on large-scale data have been published previously [8].

**Table 1:** Average processing time [s]

Input file	Clinical annotation	Network generation	Overall
VCF	45.48	58.19	91.83
VCF + TSV	177.38	203.38	352.36
Overall	103.19	121.71	205.81

## Conclusion

The individual nature of the genomic alterations causing cancer directly implies a personalized, or at least stratified approach to treating cancer if the underlying alterations are known. With the rapid drop in sequencing cost, sequencing has become routine for most cancers, but the interpretation of this data is still a major hurdle to clinical implementation of personalized oncology.

With PeCaX we present a novel tool for the exploration of the mutational landscape of a cancer patient and for treatment hypothesis generation, e.g. in the context of Molecular Tumor Boards. It is deployed in Docker containers guaranteeing full reproducibility independent of the operating system and as it is a local application it ensures data security and privacy. A local results database is used to keep track of the results and notes taken by the user. The combination of clinical variant annotation, gene drug interaction networks visualizing somatic variants in their pathway context and interactive web-based visualizations makes PeCaX unique and ensures ease of use for all users without the requirement of programming experience. Its service-oriented architecture, the front end, the graph database in the back end, and the interactive graph visualization components constitute significant developments and in their combination in PeCaX a significant advancement over the mere tabular annotation of somatic variants. PeCaX supports the diagnostic workflow as conducted in Molecular Tumor Boards, to reach transparent personalized therapeutic decision in a shorter amount of time.

In the future, we plan to allow the upload of multiple VCF files at once for an easier comparison between patients. The comparison between patients and screening for possible treatment could be further improved by the integration of gene expression values. Standard VCFs do not contain gene expression values. However, this information might be added to PeCaX as the visualization of those values is already available in BioGraphVisart as a standalone tool. Moreover, the information content for cancer diagnosis and targeted therapy could be enriched by integrating a gene fusion detection algorithm in the clinical variant annotation as fusion genes have a tumor-specific expression. PeCaX might also include information on the patient’s background provided by the user, e.g., gender, age, and previous therapies, to prioritize possible treatments based on demographic limitations. Additionally, a quantitative usability study needs to be performed to confirm the added value for the interpretation of gene alterations, especially in the context of Molecular Tumor Boards.

## Availability and Requirements

- **Project name:** PeCaX
- **Project home page:** https://github.com/KohlbacherLab/PeCaX-docker
- **Demo website:** We provide a web page with example data for demonstration purpose only at https://pecax.informatik.uni-tuebingen.de.
- **Operating system:** Platform-independent via Docker containers
- **Programming language:** Javascript
- **Other requirements:** Docker Engine release 1.13.0 or higher, Compose release 1.10.0 or higher
- **License:** MIT
- **Any restrictions to use by non-academics:** Some functionality requires a valid license for the KEGG pathway database for commercial use.

## Supplementary Information

**Additional file 1.** Components, Installation & Availability, Use Case, Performance.

## Data Availability

The source code of PeCaX is available in the KohlbacherLab repository, https://github.com/KohlbacherLab/PeCaX-docker.

## Abbreviations

NGS: Next-generation sequencing
SNV: Single-nucleotide variant
CNV: Copy number variation
VCF: Variant call format
GUI: Graphical user interface
MTB: Molecular tumor board
VEP: Variant effect prediction
COSMIC: Catalogue of somatic mutations in cancer
CGI: Cancer genome interpreter
KEGG: Kyoto Encyclopedia of genes and genomes
