# Supplementary material for: The personalized cancer network explorer (PeCaX) as a visual analytics tool to support molecular tumor boards
Source: BMC Bioinformatics. 2023 Mar 8;24:88. doi: 10.1186/s12859-023-05194-3 (PMC9993744; doi:10.1186/s12859-023-05194-3)
Supplement: Supplementary file 1 — Additional file 1. Components, Installation & Availability, Use Case, Performance. [file 12859_2023_5194_MOESM1_ESM.pdf]

PeCaX

01.11.2021

Contents

1 Introduction **1**

2 Components **1**
2.1 Clinical Annotation Pipeline . . . 2
2.1.1 Input & Parameters . . . 2
2.2 Network Generation . . . 2
2.3 Interactive Graphical User Interface . . . 3
2.3.1 ClinVAP Report . . . 3
2.3.2 Interactive Network Visualization . . . 5
2.4 Data Management . . . 5

3 Implementation & Availability **5**
3.1 Demo-Version . . . 5
3.2 Usage with Docker . . . 5
3.2.1 Requirements . . . 6
3.2.2 Availability . . . 6
3.2.3 Implementation . . . 6

4 Software Availability **13**

5 Use case **14**

6 Performance **14**

1 Introduction

PeCaX, Personalized Cancer and Network Explorer, is a visual analytics tool for the identification of patient specific cancer mechanisms by providing a complete mutational profile from variants to networks. It employs ClinVAP to perform clinical variant annotation which focuses on processing, filtering and prioritization of the variants to find the disrupted genes that are involved in carcinogenesis and to identify actionable variants from the mutational landscape of a patient. In addition PeCaX creates networks showing the connections between the driver genes and the genes in their neighbourhood using pathway resources (SBML4j). Its interactive visualisation (BioGraphVisart) supports easy network exploration and node grouping by related pathways.

2 Components

The application consists of four major components: the clinical variant annotation pipeline, the network generation, the interactive graphical user interface (GUI), and the data management.

2.1 Clinical Annotation Pipeline

Clinical Variant Annotation Pipeline (ClinVAP) generates concise case reports from long list of somatic mutations given inn VCF-format by first annotating them functionally and clinically, then by prioritizing and filtering them based on their variant effect together with clinical actionability (for more detail see `https://doi.org/10.1093/bioinformatics/btz924`).

The functional annotation includes the prediction of the mutation effect on protein level and the priorization of the variants based on their effect or importance. The functional variant annotation is done with the Ensemble Variant Effect Predictor in offline mode to ensure data privacy. VEP plugins SIFT and PolyPhen are used to predict variant effect on protein. Variants are filtered based on passing quality measures of the NGS pipeline used to generate VCF files, their predicted effects by VEP, SIFT and PolyPhen. The remaining variants are passed to the reporting application.

The clinical annotation includes the selection of clinically relevant variants or mutated genes together with the targeting therapeutics mechanistically or with the clinical evidence. The ClinVAP uses background knowledgebases developed in-house for the clinical annotation. It is created by integrating publicly available databases to contain information of driver gene annotation, clinically relevant known pharmacogenomics effects information, adverse effect information, mechanistic drug targets. The database is queried with observed variants to identify therapeutics with known effects directly on the variants, and with the gene name to identify driver genes and the therapeutics with known effect on the disrupted genes. It also enables users to filter results more providing the diagnosis information as ICD10 code format. It provides one more layer of prioritization by selecting the results associated with the provided cancer type. Evidence level is also calculated for the results to show the significance of the associations between reported targets and drugs and their observed effect.

After completing variant and clinical annotation, the ClinVAP Report is shown in the GUI and it can be downloaded as JSON or PDF formats.

2.1.1 Input & Parameters

The GUI to start an analysis is shown in Fig. 1. A mandatory Variant Call Format (VCF) file containing somatic variant (SNV) will be analysed and annotated with ClinVAP. The results will be structured in a report (.json-format) and displayed in interactive tables (ClinVAP Report) once the pipeline is finished. An example file can be found here: `https://raw.githubusercontent.com/KohlbacherLab/PeCaX-docker/main/test_files/lung.vcf`. Please make sure the file you upload matches the standard VCF-format as given in the example.

In addition to the SNV information, CNV information can be analyzed and annotated. They can be given in an optional .tsv-file. The file has to have the same column names as in the example file here: `https://raw.githubusercontent.com/KohlbacherLab/PeCaX-docker/main/test_files/lung.tsv`. Please make sure the file you upload matches the standard TSV-format as given in the example.

The human genome assembly version that was used in the original NGS pipeline while calling the variants needs to be given. The default value is GRCh37, alternatively GRCh38 can be selected.

ClinVAP can analyse the SNV information according to a diagnosis. This should be entered as an ICD10 code. The diagnosis can be used as filter:

- sort: Option to sort results based on their diagnosis similarity score.
- filter: Option to only get the results of a certain cancer type given as diagnosis.
- sort, filter, prioritize: Option to get sorted results for evidence level A, B and C, filtered results for evdence level D and E.

2.2 Network Generation

For the genes in the ClinVAP report, PeCaX creates networks of those genes, their neighbouring genes and targeting drugs with SBML4j. SBML4j is a service for persisting biological models and pathways in SBML format in a graph database. It is written in Java as a Spring Boot Application and the data is stored in a neo4j graph database instance.

The biological models are integrated into one unified knowledge graph from which network mappings are created. There are four types of network mappings available depending on the given models, regulatory, signalling, protein-protein-interaction and metabolic mappings. Those mappings can then be explored, annotated and searched in. A

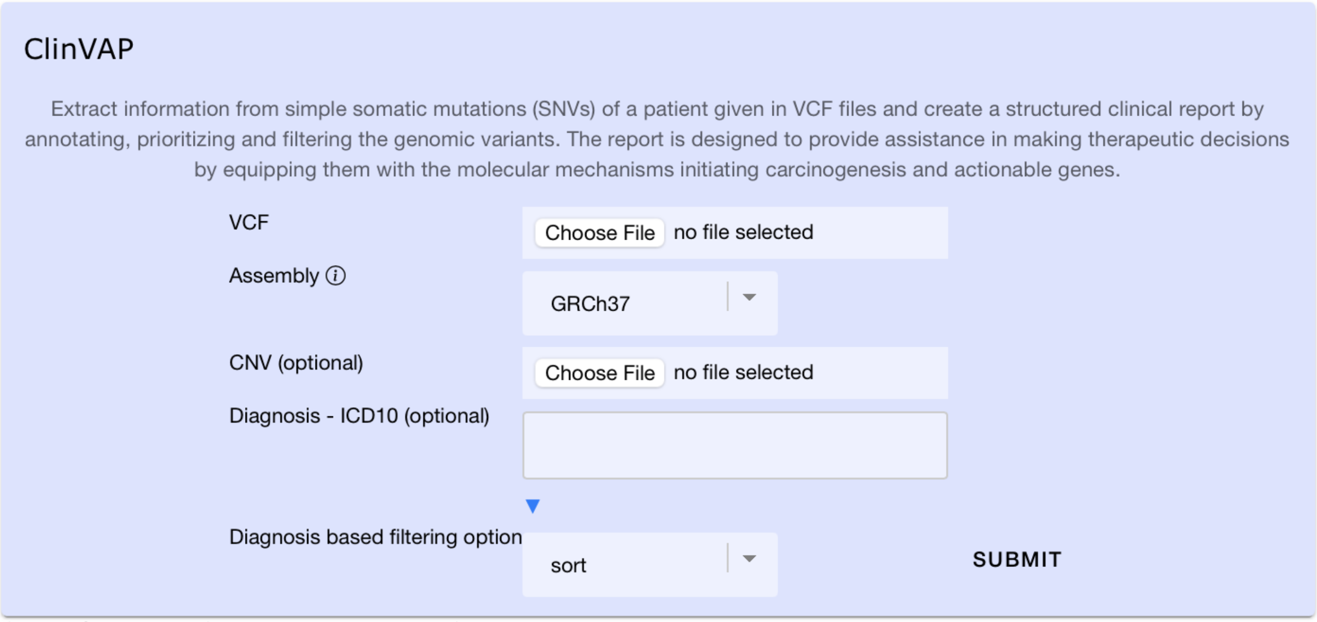

Figure 1: Interface to start the analysis. A new analysis can be configured and submitted by uploading a VCF-file and selecting the correct assembly. The upload of a TSV-file, selction of a diagnosis by its ICD10 code and filtering with it are optional.

user can run graph algorithms on the mappings and retrieve the created subgraphs in the GraphML format. A client can annotate a mapping with arbitrary data on nodes and relationships.

A REST interface is provided to interact with the pathways as well as with the network mappings. Communication with the REST API is JSON based and networks are provided in the GraphML format. The full documentation of the API can be found at Swaggerhub `https://app.swaggerhub.com/apis-docs/tiede/sbml4j/1.1.7`.

SBML4j also offers methods to filter a network by type of node or relationship and individual entities. For one or multiple named nodes (i.e. genes) a network context can be calculated which is stored as a separate network. This enables a user to get a representation of the network surroundings of a gene of interest or get the up- and/or downstream genes in the given models across standard pathway boundaries. With multiple supplied nodes the same query will calculate a minimal network containing all the given genes, provided they have a known network context.

PeCaX sends the list of genes of every table generated by ClinVAP to SBML4j to generate a minimal network. All genes that are provided in this way are annotated with a boolean value, indicating that those genes are present in the table and to differentiate these genes from those that are added by the algorithm performed by SBML4j. Additionally the type of driver gene is added to the respective nodes in the network if they are known.

The SBML4j database used in PeCaX is built from 61 cancer-related pathways from the KEGG pathway database. The pathways are integrated with each other and a models-spanning network mapping is created which is used as the basis for the presented networks. To not miss any interaction from using one specific network mapping type, all interactions and relations are included for this base-network. This network additionally enriched with drug-target information gathered from the list of approved drugs that are freely available from Drugbank (https://drugbank.ca).

2.3 Interactive Graphical User Interface

2.3.1 ClinVAP Report

The results of the annotation and analysis with ClinVAP are represented in interactive tables. If information from external sources is available for a gene in the table, the related web links are listed next to the gene name in a drop-down menu. Next to each table, the related gene network is displayed, if applicable. Each table can be downloaded as as PDF-file. Fig. 2 shows example tables on the left. The tables contain information about:

- Somatic Mutations in Known Driver Genes: List of cancer driver genes along with the mutations observed in the patient. Consequence column provides the predicted effects of the variants on the protein sequence.

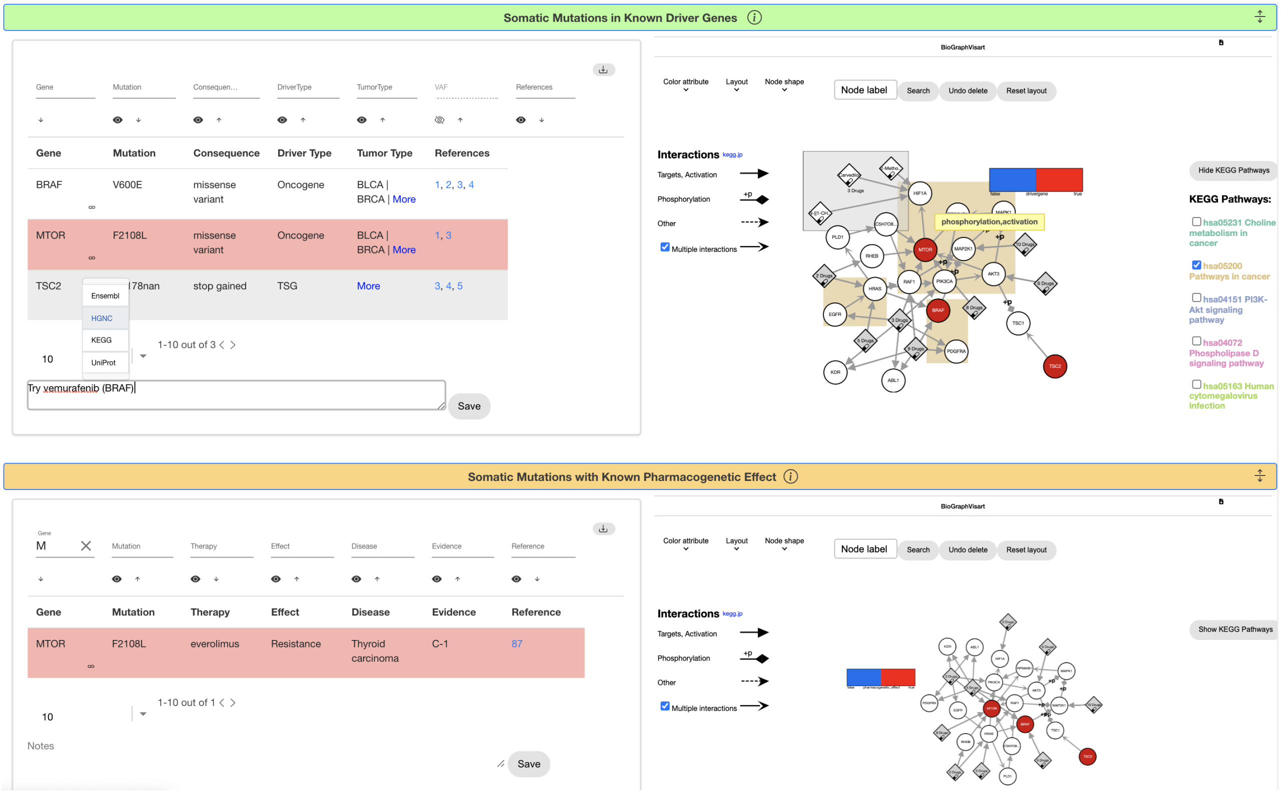

Figure 2: Screenshot of two tables (left) and related networks (right).

Tumor type column gives the list of cohorts in which the gene is identified as driver. VAF (variant allele frequency) column shows the proportion of the variant allele to the coverage of that loci. Reference column represents the driver gene sources that catalogued the corresponding gene as driver. Driver gene information is obtained from Vogelstein et. al, Uniprot, TSGene, IntoGen and COSMIC.

- Somatic Mutations with Known Pharmacogenetic Effect: List of drugs with the evidence of targeting the observed variant of the mutated gene, and the documented drug response for the given mutational profile. Evidence level letter represents: A = validated association, B = clinical evidence, C = case study, D = preclinical evidence, E = inferential association. Evidence level number represents the matching type between the observed variant and the database result: 1 = same variant, 2 = different variant, same consequence, 3 = different variant, different consequence, same gene. The information is obtained from CIViC, CGI and DrugBank.

- Somatic Mutations in Pharmaceutical Target proteins: Pharmacogenomics Summary of Drugs Targeting Affected Genes Therapies that have evidence of targeting the affected gene. Evidence level letter represents: A = validated association, B = clinical evidence, C = case study, D = preclinical evidence, E = inferential association. Evidence level number represents the matching type between the observed variant and the database result: 1 = same variant, 2 = different variant, same consequence, 3 = different variant, different consequence, same gene. The information is obtained from CIViC, CGI and DrugBank.

- Summary of Cancer Drugs Targeting Affected Genes:: List of cancer drugs targeting the mutated gene. Information is obtained from DrugBank, Therapeutic Target Database, IUPHAR, and Santos et al.

- Adverse Effects: List of drugs with known adverse effects on observed variant and disrupted genes.

- References: The publications of the reference IDs given in the tables.

- Appendix: All the somatic variants of the patient with their dbSNP and COSMIC IDs.

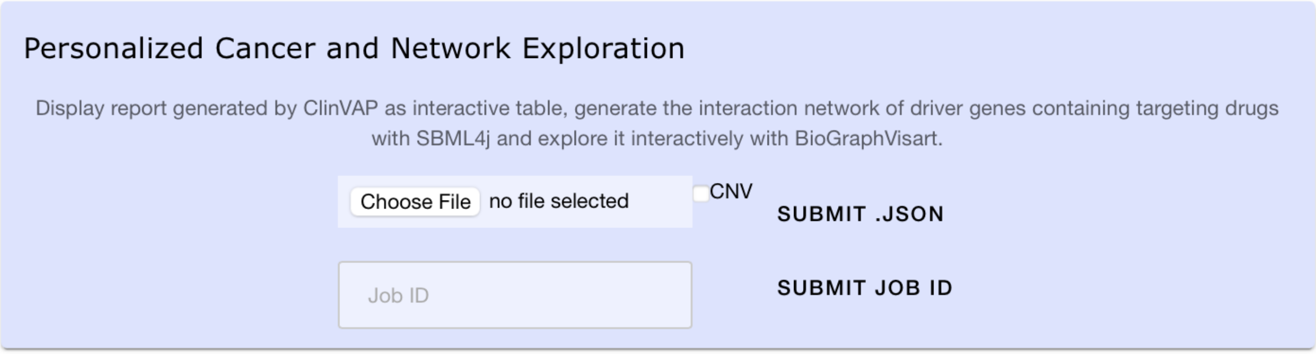

Figure 3: Interface to restore previous jobs by uploading the according JSON-file or entering the job id.

2.3.2 Interactive Network Visualization

PeCaX integrates the web-based tool BioGraphVisart for the interactive network visualization. BioGraphVisart receives the network from SBML4j as a GraphML-file via REST API calls and displays it. Fig. 2 shows example networks on the right.

The nodes of the network represent the genes and related drugs targeting those genes. If multiple drugs have the same gene as target, they are collapsed into one squared node, which can be extended by clicking on it. The nodes are coloured according to the information if they are stored in the related table. For the table Somatic Mutations in Known Driver Genes the driver genes in the network are additionally annotated with information of what driver type they are of, which is displayed when the mouse is moved over a node.

The edges between nodes represent the interaction(s) between two genes or a drug and a gene. The type of interaction can be seen in the interaction legend or by moving the mouse over an edge of interest. Multiple edges between two nodes are by default collapsed into one. This can be disabled in the interactions legend.

The most common KEGG Pathways for the displayed genes can be calculated and the according genes can be grouped by them, highlighted by coloured squares.

The network can be downloaded in the file formats PNG, SVG for presentations or GraphML for further investigation. BioGraphVisart can also be used as standalone application with port 3000.

2.4 Data Management

For each uploaded VCF file a new database entry is created containing information about the user-selected project name, a unique job ID, parameters set for the clinical variant annotation, IDs of the networks generated and stored in SBML4j, and the information contained in the tables displayed on the front end.

The results of a completed job can be accessed by the job id again (Fig. 3). In addition, the report of a analysed vcf-file can be downloaded as a json-file. This file can be loaded again and the contained tables are displayed.

3 Implementation & Availability

3.1 Demo-Version

A demo-version of PeCaX is available at `https://pecax.informatik.uni-tuebingen.de`. It offers nine different vcf-files, seven with an additional tsv-file. Since real patient data is highly sensitive and it is illegal to make it public, we compiled example datasets from the publicly available data source Cancer Genome Interpreter (CGI) biomarkers dataset. We separated the biomarkers based on the cancer type they were observed with. Then, we created individual VCF files for SNVs and TSV files for CNVs using the information given in the biomarkers data file for specific cancer types. This demo-version provides all functionality of the full version of PeCaX and demonstrates the accessibility by multiple users using a local installation on a server with a publicly accessible address.

3.2 Usage with Docker

The application consists of eight Docker images for the clinical annotation pipeline, the network database, the network visualization, and data management.

The images and related volumes are orchestrated via docker-compose. Volumes created by docker compose are prefixed with the folder name where the docker-compose.yaml file resides that created the services (i.e. pecax-docker). They are briefly described here:

- *sbml4j_neo4j_vol*: used to store the data needed for the network database (config, logs, plugins, database).
- *sbml4j_service_vol*: used to store log files generated by the sbml4j service.
- *arangodb_data_container*: database directory to store the collection data (username, jobid, json, network uuids)
- *arangodb_apps_data_container*: apps directory to store any extensions

3.2.1 Requirements

- Docker Engine release 1.13.0+
- Compose release 1.10.0+
- 55 GB of physical empty space on Docker Disk Image
- Availability of the ports 3000, 3030, and 8080, i.e. not being used by other application.

3.2.2 Availability

All images are publicly available on Docker Hub `https://hub.docker.com/orgs/pecax/repositories`. The application orchestrates eight images via docker-compose which are:

- Main image, *pecax*: Orchestrates the file transfers between the services and offers the GUI.
- Data management image, *arangodb*: stores the job collections in an ArangoDB database
- *clinvap_api_flask*:
- *clinvap_api_nginx*:
- *clinvap_api_file_deploy*:
- *clinvap_api_nextflow*:
- *sbml4j*: Creates networks based on input-gene lists and annotation data. Provides networks in GraphML format.
- *neo4j*: Database for storing the networks and calculating the gene-neighbourhoods using the APOC (https://neo4j.com/deve apoc/) plugin.
- Network visualization image, *biographvisart*: Process a GraphML file from SBML4j and visualize it interactively.

3.2.3 Implementation

To run the pipeline for the first time, please follow the steps described next.

1. Clone the Git repository via:

   ```
   git clone https://github.com/KohlbacherLab/PeCaX-docker.git
   ```

   This will also download local folders that are used in the following ways:

   - The local folder *db_backups* is used by the sbml4j.sh script to store the network database backups. See instructions below for details.
   - The local folder *scripts* is used by the sbml4j.sh script to store the scripts needed for setting up the database volume.

- The local folder *conf* is used by the sbml4j.sh script to store the configuration file needed for the neo4j database. Any change you make to this configuration file will only be activated on recreating the volumes for the sbml4j service and database.

2. Change to the cloned directory

```
cd PeCaX-docker
```

3. **Setting up ClinVAP**

   Download the assembly:

   - For human genome assembly GRCh37, use:

     ```
     docker-compose up vep_files_GRCh37
     ```

     Afterwards, to free up space, remove the downloaded image:

     ```
     docker rmi bilges/clinvap_file_deploy:vP_GRCh37
     ```

   - If your analysis requires GRCh38, use:

     ```
     docker-compose up vep_files_GRCh38
     docker rmi bilges/clinvap_file_deploy:vP_GRCh38
     ```

   The assemblies can be served in parallel and need to be downloaded only once as long as the volume pecax-docker_clinvap_downloads is not removed.

4. **Setting up SBML4j**

   4.1 Preparation

   4.1.1 Accessibilty of the SBML4j Service in the PeCaX ecosystem:
   For security reasons the SBML4j service is not exposed to the host machine, so to be able to directly interact with SBML4j you will need to temporarily change the docker-compose.yaml file. In the service block "sbml4j" you need to change

   ```
   expose:
   - "8080"
   ```

   to

   ```
   ports:
   - "8080:8080"
   ```

   Once you are finished with creating and setting up your desired network database you are advised to revert this change.

   4.1.2 Communicating with the SBML4j service
   The following instructions provide examples for the communication with the REST interface of SBML4j using CURL and Python. Alternatively you can use a tool of your choice to issue GET and POST http requests to the SBML4j service, like Postman. You can find the API definition for initialising the requests in your tool of choice at `https://github.com/kohlbacherlab/sbml4j-compose/api_doc/sbml4j.yaml`
   Please note, that file-names, argument values and UUIDs used are only exemplary and need to replaced with the actual values from your installation. Also note, that the " character in the examples below is used to signify line breaks to make the blocks more readable and might need to be removed before executing the snippets, depending on your system.
   To use the Python code examples you need to install the *pysbml4j* Python package:

   ```
   pip install pysbml4j
   ```

Then use it in your Python environment of choice with:

```
import pysbml4j

client = pysbml4j.Sbml4j()
```

If you are not running the service on you local system, you need to configure pysbml4j accordingly:

```
import pysbml4j
from pysbml4j import Configuration

client = pysbml4j.Sbml4j(
    Configuration("http://mysbml4jhost:8080"))
```

We will need to store uuids of pathways created from the uploaded SBML models. In the python examples below we will use the following list variable for this:

```
pathwayUUIDs = []
```

For more details see the pysbml4j documentation at `https://github.com/kohlbacherlab/pysbml4j`.

4.2 Initialize the docker volumes needed using the provided script

To initialize the volumes used for the network database and the SBML4j service use the sbml4j.sh script. Inside the main directory (default: PeCaX-docker) run:

```
./sbml4j.sh -i
```

to install all prerequisites for the SBML4j service and its database. If your working directory (where you run the docker-compose commands) is named differently from PeCaX-docker (case insensitive) your volumes will get this directory name (in lower case format) as prefix.

If you need to change the prefix (because you intend run the docker-compose command in a different folder than this script), you need to add the option

```
-p my_prefix
```

to the above call to make sure that the volumes are prefixed with the correct name. In case you forgot, you can remove the previously created volumes and rerun the above command with the additional -p option.

4.3 Selecting a source

The demo version of PeCaX accessible at `https://pecax.informatik.uni-tuebingen.de` uses a selection of 61 pathway maps from the KEGG pathway database. If you want to recreate this version of PeCaX in your local environment, follow steps 3 and 4 below to download and translate the KEGG pathways used. If you want to use different source models head over to `https://github.com/kohlbacherlab/sbml4j` to learn about the necessary details to look for when using SBML models with SBML4j for non-metabolic network-mappings.

4.4 Get the KEGG pathway files

List 1 shows the pathway identifiers of the KEGG pathways used in this publication. KEGG provides their own markup language files for their pathways. You can download these kgml files directly from their website (kegg.jp) or through their API. Make sure you understand the license requirements before starting the download (see `https://www.kegg.jp/kegg/rest/` for details).

4.5 Translate pathway files

In order for SBML4j to be able to process the KEGG pathway models they need to be translated to the SBML format. We used the KEGGtranslator version 2.5 [1] for this. Please find more info on KEGGtranslator here: `http://www.cogsys.cs.uni-tuebingen.de/software/KEGGtranslator/`. Go to `http://www.cogsys.cs.uni-tuebingen.de/software/KEGGtranslator/downloads/index.htm` and download the version 2.5 executable jar file, which you can run using your local java runtime installation. We used the following command line options for translating the pathway maps in addition to providing input and output directories for the kgml and sbml files respectively:

```
--format SBML_CORE_AND_QUAL
--remove-white-gene-nodes TRUE
--autocomplete-reactions TRUE
--gene-names FIRST_NAME
--add-layout-extension FALSE
--use-groups-extension FALSE
--remove-pathway-references TRUE
```

4.6 Upload models to SBML4j

You need a running SBML4j service for the next steps to complete. To get that run:

```
docker-compose up sbml4j
```

Once you see the message

```
Started Sbml4jApplication in x.xxx seconds
```

the service is up an running and you can issue http request to the exposed API. By issuing a POST request to the /sbml endpoint one or multiple SBML formatted xml files can be uploaded to SBML4j. For best performance we recommend uploading the model files one by one or in small chunks of 5 models or less. Choose the same organism, source and version parameters for all pathway maps to ensure proper integration in the next step. For details on the RESTful interface visit `https://app.swaggerhub.com/apis-docs/tiede/sbml4j/1.1.7`

An exemplary curl command for uploading SBML models to a local service is shown below (you can upload multiple files at once by providing multiple -F files=@ parameters to the curl command, but can also just use one at a time) :

```
curl -v \
 -F files=@/absolute/path/to/sbml/model/file1.xml \
 -F files=@/absolute/path/to/sbml/model/file2.xml \
 -F "organism"="hsa" \
 -F "source"="KEGG" \
 -F "version"="97.0" \
 -o response.file \
http://localhost:8080/sbml4j/sbml
```

We redirect the response of the service here into the file 'response.file' using the '-o' option. This file will then contain a json-formatted response containing basic information about the uploaded model(s). Be sure to at least save the provided 'uuid' (or 'UUID', they are identical) for each model as we will need those later.

Using the pysbml4j package uploading SBML models with python is as easy as:

```
resp = client.uploadSBML(
    [/absolute/path/to/sbml/model/file1.xml,
    /absolute/path/to/sbml/model/file2.xml],
    "hsa",
    "KEGG",
    "97.0")
print( "The UUID of pathway in file1.xml is {}, of file2.xml it is {}"
  .format(resp[0].get("uuid"), resp[1].get("uuid")))
pathwayUUIDs.add(resp[0].get("uuid"))
pathwayUUIDs.add(resp[1].get("uuid"))
```

Please note that the files provided need to be in a list, even when uploading only a single file as is shown here:

```
resp = client.uploadSBML([/absolute/path/to/sbml/model/onlyfile.xml], "hsa", "KEGG",
"97.0")
```

4.7 Create pathway collection

A network mapping always refers to one pathway instance in the database. In order to build network mappings for multiple KEGG pathways we combine all entities, relations and reactions in a collection

pathway element which can be used subsequently to generate network mappings. The endpoint /pathwayCollection accepts a POST request with a JSON formatted body containing the elements: name, description and sourcePathwayUUIDs. Name and description are used as pathwayIdString and pathwayDescription respectively. The field sourcePathwayUUIDs has to be an array of character strings, each string being one UUID of a pathway that shall be added to the collection element.

```
curl -v \
 -H "Content-Type: application/json" \
 -d '{"name":"BMC_Collection", \
      "description": "This is the Collection for the BMC Publication",\
      "sourcePathwayUUIDs":["909520db-8ca9-40df-bffe-af9e48e93c48", \
            "9d959b42-f1da-4061-960b-4b58e1ba3c16"]}' \
 -o response.pwcoll \
http://localhost:8080/sbml4j/pathwayCollection
```

A simple python call making use of pysbml4j can look like this:

```
collUUID = client.createPathwayCollection("KEGG61-97.0",
    "Collection pathway for all 61 KEGG pathways", pathwayUUIDs)
print(collUUID)
```

The endpoint returns the UUID of the created collection pathway, which can be used in the following calls to create the network mappings.

4.8 Create network mappings

To create the network mappings from a pathway a POST request to the /mapping endpoint has to be issued. The UUID of the pathway is part of the URL as can be seen here:

```
curl -v \
 -d "mappingType"="PATHWAYMAPPING" \
 -d "networkname"="PWM-KEGG-BMC" \
 -o response.mapping \
http://localhost:8080/sbml4j/mapping/b6da7dc5-4dc4-4991-85c0-5ab75e2bf929
```

```
resp = client.mapPathway(collUUID, "PATHWAYMAPPING", "PWM-KEGG_BMC")
print("The created mapping has the uuid:{}".format(resp.get("uuid")))
```

The last part of the url (b6da7dc5-4dc4-4991-85c0-5ab75e2bf929) is the collUUID generated in the previous step. Be sure to fill in the UUID of your installation when creating the pathway collection. The UUIDs are generated by SBML4j and will differ every time you run this procedure.

The artifical mapping type 'PATHWAYMAPPING' can be used to not restrict the elements or relations being mapped and will map every entity, relation and reaction into a network mapping instance. Such a network mapping has been used for PeCaX to allow for the most broad view on the network context of the genes of interest.

4.9 Prepare the Drugbank csv file

You can find the drugtarget information used in PeCaX at: `https://go.drugbank.com/releases/latest#protein-identifiers`. You will need a free account on drugbank.ca to gain access to this file, which is released under the 'Creative Common's Attribution-NonCommercial 4.0 International License.' You will have to agree to these terms and conditions to continue with the next steps described here. We used the 'Drug target identifiers' file for all approved drug groups to get a broad view on available and possible drugs and the genes and geneproducts they target. This was combined with the DrugBank Vocabulary to map the DrugBankID of drugs to their name, availavle at: `https://go.drugbank.com/releases/latest#open-data`. In order to reproduce the results found in the publication two preprocessing steps need to be performed:

Filter out all rows that are not targeting genes in Humans (column 'Species'). Consolidate rows with the same 'ID' into one row, combining the elements in the 'Drug IDs' of all those rows into one. Combine it with the DrugBank Vocabulary Here we provide an exemplary R script to perform Step 2 above:

```
# create a csv containing one entry per drug targeting a human gene
```

```
# load genes with associated drugs
genes <- read.csv("all.csv", header=TRUE, stringsAsFactors = FALSE)
genes <- genes[order(genes$Name),]

# load dictionary for drugbank id and drug name
drugbank_vocabulary <- read.csv("drugbank_vocabulary.csv", header=TRUE,
    stringsAsFactors = FALSE)

# create new dataframe
filtered_genes <- data.frame(matrix(ncol = 2, nrow = 0))
colnames(filtered_genes) <- c("Gene.Name", "Drug.IDs")

i <- 1
j <- 2

drugids <- genes[i,"Drug.IDs"]

# filter for human genes and only have one entry per gene
while(j<=nrow(genes) && i <= (nrow(genes)-1)) {
    gene1 <- genes[i,"Gene.Name"]
    gene2 <- genes[j,"Gene.Name"]
    species1 <- genes[i,"Species"]
    species2 <- genes[j,"Species"]
    if( gene1 == gene2 && species1 == "Humans" &&
        species2 == "Humans"){
        drugids <- paste(drugids, as.character(genes[j,"Drug.IDs"]),
            sep=";")
        j <- j+1
    }
    else{
        if(species1 == "Humans" && (is.na(genes[i,"Gene.Name"]) |
            nchar(genes[i,"Gene.Name"]) > 0)){
            filtered_genes[nrow(filtered_genes) + 1,] =
            list(genes[i,"Gene.Name"],drugids)
        }
        i <- j
        j <- j+1

        drugids <- genes[i,"Drug.IDs"]
    }
}

# merge drug common names and drug infos with associated genes
drugcsv <- data.frame(matrix(ncol = 8, nrow = 0))
colnames(drugcsv) <- c(colnames(drugbank_vocabulary), "Gene.Name")
for(row in 1:nrow(filtered_genes)){
    gene <- filtered_genes[row, "Gene.Name"]
    drugids <- unlist(strsplit(filtered_genes[row, "Drug.IDs"], ";"))
    for(drug in drugids){
        drug <- gsub("\\s", "", drug)
        drugcsv[nrow(drugcsv) + 1,] = c(
            drugbank_vocabulary[drugbank_vocabulary$DrugBank.ID==drug,],
            gene)
    }
}
```

```
# save new csv
write.csv(drugcsv,"drug_genes_approved.csv", row.names = FALSE)
```

4.10 Add the Drugbank csv file to the network mappings

Using the csv upload functionality of SBML4j arbitrary data can be annotated onto network nodes. The endpoint expects a 'type' parameter, giving a character string describing the type of annotation that is added, in our example the term 'Drugtarget' is used, as the csv marks every gene symbol given as a drug target for the provided list of 'Drug IDs'.

Please note, that since there can be multiple Drugs targeting the same gene or gene-product, the annotation-names will include a numbering scheme in addition to the column names given in the csv file.

Make sure to set the 'networkname' to "PeCaX-Base" (case-sensitive). SBML4j for PeCaX is configured to use the network with this name as basis for calculating the networks by default. If you want to use a different name, make sure to also change the appropriate config parameter in the 'docker-compose.yaml' file.

You can use the curl command to upload a csv file and annotate the created network mapping with the contained data:

```
curl -v \
 -F upload=@drug_genes_approved.csv \
 -F "type"="Drugtarget" \
 -F "networkname"="PeCaX-Base" \
 -o response.drugbank \
http://localhost:8080/sbml4j/networks/a68645cb-f3bb-49d3-b05f-7f6f05debba3/csv
```

The uuid in the url (here a68645cb-f3bb-49d3-b05f-7f6f05debba3 as example) is the uuid of the PATHWAYMAPPING created in Step 7. Create network mappings and can be found in the response.mapping file created in that section using the curl command. Be sure to replace the uuid shown here with your own uuid as it is specific to your database.

The python package also offers this functionality:

```
net = client.getNetworkByName("PWM-KEGG-BMC")
net.addCsvData("drug_genes_approved.csv", "Drugtarget", networkname="PeCaX-Base")
```

Now your installation of PeCaX should contain the same base network-database that can be found in the demo-version at `https://pecax.informatik.uni-tuebingen.de`

4.11 Save the network database to reset your networks in PeCaX in the future

Before backing up the network database you need to stop the service with

```
docker-compose down
```

Then you can use the provided script to backup the database:

```
./sbml4j.sh -b pecax-base
```

This will create two '.dump' files in the db_backups folder containing the database backup you just created. For security reason it is advised to reset the port setting for the sbml4j service as described in step 4.1.1. Make sure to backup your database dumps at a save location for later reference.

5. Start PeCaX services via

```
docker-compose up pecax
```

6. In Browser of your choice open localhost:3030. We recommend using full screen to enjoy the full experience.

7. Exit and terminate PeCaX pressing ctrl+c and enter

```
docker-compose down
```

Restoring the state of the database The networks are stored in a docker volume and are thus persisted between individual PeCaX sessions. If you however delete or prune your docker volumes, while the service is not running, the network volume will be deleted and you will have to restore the database. Before restoring the network database you need to stop the service with

```
docker-compose down
```

Then you can revert your database back to the previously saved state by using:

```
./sbml4j.sh -r pecax-base
```

4 Software Availability

The source code of PeCaX is open source under MIT license and available on `https://github.com/KohlbacherLab/PeCaX-docker`. If you would like to contribute, you may

- open an issue on GitHub,
- fork the repository, make changes and submit a pull request for us to review the changes and merge your contribution.

Please contact us on mirjam.figaschewski@uni-tuebingen.de for further information/help.

Listing 1: KEGG Pathway Maps used in the demo version

```
hsa03320 PPAR signaling pathway
hsa04010 MAPK signaling pathway
hsa04012 ErbB signaling pathway
hsa04014 Ras signaling pathway
hsa04015 Rap1 signaling pathway
hsa04020 Calcium signaling pathway
hsa04022 cGMP-PKG signaling pathway
hsa04024 cAMP signaling pathway
hsa04060 Cytokine-cytokine receptor interaction
hsa04064 NF-kappa B signaling pathway
hsa04066 HIF-1 signaling pathway
hsa04068 FoxO signaling pathway
hsa04070 Phosphatidylinositol signaling system
hsa04071 Sphingolipid signaling pathway
hsa04072 Phospholipase D signaling pathway
hsa04080 Neuroactive ligand-receptor interaction
hsa04110 Cell cycle
hsa04115 p53 signaling pathway
hsa04150 mTOR signaling pathway
hsa04151 PI3K-Akt signaling pathway
hsa04152 AMPK signaling pathway
hsa04210 Apoptosis
hsa04218 Cellular senescence
hsa04310 Wnt signaling pathway
hsa04330 Notch signaling pathway
hsa04340 Hedgehog signaling pathway
hsa04350 TGF-beta signaling pathway
hsa04370 VEGF signaling pathway
hsa04371 Apelin signaling pathway
hsa04390 Hippo signaling pathway
hsa04510 Focal adhesion
hsa04512 ECM-receptor interaction
hsa04520 Adherens junction
hsa04630 JAK-STAT signaling pathway
```

```
hsa04915 Estrogen signaling pathway
hsa05200 Pathways in cancer
hsa05202 Transcriptional misregulation in cancer
hsa05203 Viral carcinogenesis
hsa05204 Chemical carcinogenesis
hsa05205 Proteoglycans in cancer
hsa05206 MicroRNAs in cancer
hsa05210 Colorectal cancer
hsa05211 Renal cell carcinoma
hsa05212 Pancreatic cancer
hsa05213 Endometrial cancer
hsa05214 Glioma
hsa05215 Prostate cancer
hsa05216 Thyroid cancer
hsa05217 Basal cell carcinoma
hsa05218 Melanoma
hsa05219 Bladder cancer
hsa05220 Chronic myeloid leukemia
hsa05221 Acute myeloid leukemia
hsa05222 Small cell lung cancer
hsa05223 Non-small cell lung cancer
hsa05224 Breast cancer
hsa05225 Hepatocellular carcinoma
hsa05226 Gastric cancer
hsa05230 Central carbon metabolism in cancer
hsa05231 Choline metabolism in cancer
hsa05235 PD-L1 expression and PD-1 checkpoint pathway in cancer
```

5 Use case

For the preparation of an MTB case, the user logs in with the project ID which is the MTB date. Afterwards, the user uploads a VCF file with SNVs and and a TSV-file with CNV information. The assembly is not changed by the user if the default option was used in the NGS-pipeline. It is known, that the patient of interest has the diagnosis with ICD10 code c341 and the user decides to filter the clinical variant annotation by it. The users starts the pipeline. The user waits for the pipeline to finish. Once finished, the results are displayed in tables and the networks are shown next to them. Afterwards, the user looks into the somatic mutations in known driver genes. The user looks up a gene in UniProt and Ensembl opened in extra windows. OnkoKB is visited for the associated mutation in an extra window. Information of interest is quoted and saved by the user. The user filters the table for a specific tumor type. The table is reduced to the genes with the selected tumor type. Afterwards, the user searches the gene in the network. The user inspects the neighborhood of the gene of interest. The user inspects a drug, that is targeting a neighbored gene inhibiting the gene of interest, on DrugBank in an extra window. The user makes notes and saves them. The user highlights a specific KEGG pathway and the genes are grouped by it. The user zooms into a part of interest and downloads it. Then the user highlights the gene row and examines the other tables with focus on this gene. The user looks into the reference given for a somatic mutation with known pharmacogenetic effect in an extra window and saves notes made. The user saves the tables in PDF and shares the PDF and saved images and/or the log-in ID and job ID with the clinicians participating in the MTB.

6 Performance

The performance was evaluated on a MacBook Pro with a 3.1 GHz Dual-Core Intel Core i5 processor and 16 GB 2133 MHz LPDDR3 memory with a local installation of PeCaX. Table 1 contains detailed information on the processing time of an analysis from submitting the data until the full report is displayed.

Data	Clinical Annotation			Network generation			Overall		
	1	2	3	1	2	3	1	2	3
any_cancer_type.vcf	36	42	43	122	140	130	158	182	173
breast_adenocarcinoma.vcf	43	46	43	23	20	20	66	66	63
chronic_myeloid_leukemia.vcf	42	50	53	10	9	9	52	59	62
colorectal_adenocarcinoma.vcf	42	42	42	48	48	50	90	90	92
cutaneous_melanoma.vcf	42	42	53	63	48	55	105	90	108
lung_adenocarcinoma.vcf	43	42	42	65	69	73	108	111	115
lung.vcf	42	52	42	22	28	30	64	80	72
non_small_cell_lung.vcf	42	42	52	16	20	25	58	62	77
thyroid_carcinom.vcf	42	52	62	17	17	20	59	69	82
any_cancer_type.vcf + .tsv	381	374	382	248	291	349	629	665	731
breast_adenocarcinoma.vcf + .tsv	283	289	296	63	82	88	346	371	384
colorectal_adenocarcinoma.vcf + .tsv	254	268	137	73	81	81	327	349	218
cutaneous_melanoma.vcf + .tsv	194	166	183	183	221	208	377	387	391
lung_adenocarcinoma.vcf + .tsv	182	183	180	64	110	95	246	293	275
lung.vcf + .tsv	208	163	172	86	133	136	294	296	308
non_small_cell_lung.vcf + .tsv	85	112	82	21	38	33	106	150	115

Table 1: Processing time [s] for the steps of clinical annotation from the time of job submission until the report is displayed, network generation from receiving the genes until the networks are displayed, and overall time from submitting a job until the full report is displayed. Data was analyzed three times.
